# Nucleic acid probe based on DNA-templated silver nanoclusters for turn-on fluorescence detection of tumor suppressor gene p53†

**DOI:** 10.1039/c8ra04716b

**Published:** 2018-07-18

**Authors:** Dan Han, Chunying Wei

**Affiliations:** Key Laboratory of Chemical Biology and Molecular Engineering of Ministry of Education, Institute of Molecular Science, Shanxi University Taiyuan 030006 P. R. China weichuny@sxu.edu.cn

## Abstract

In this paper, we construct a fluorescence nucleic acid probe based on DNA-templated silver nanoclusters (DNA-Ag NCs) for the detection of the p53 gene. The fluorescence biosensing of the “turn-on” model is successfully implemented as a result of the target-triggered configurational change in the hairpin DNA probe and the synthesis of fluorescent Ag NCs. With this biosensor, the limit of detection (LOD) for the p53 gene is 3.57 nM and the linear range is 250–2500 nM in phosphate buffer solution, while a LOD of 6.06 nM in the linear range of 250–2500 nM is obtained in 1% diluted fetal calf serum. So this probe, with its advantages of specificity, practical application, easy operation and low cost, will have favourable development prospects in biological sensing and imaging.

## Abstract 2

“Turn-on” fluorescence detection for p53 gene based on target-triggered opening of hairpin DNA probe and synthesis of DNA-Ag NCs.

## Graphical abstract

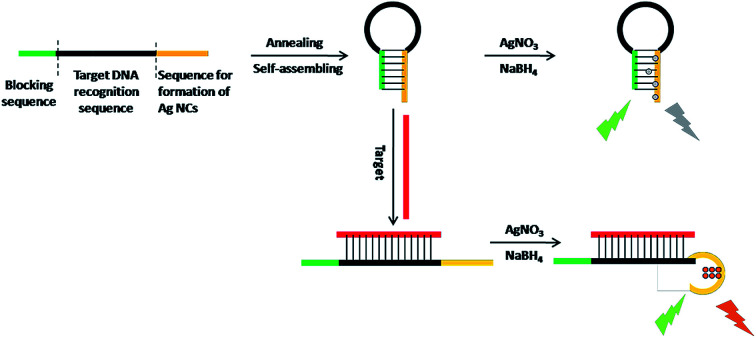

## 1. Introduction

The tumor suppressor gene p53 is the gene with the highest correlation found so far with human cancer. Protein encoded by p53 gene is a transcription factor that is responsible for DNA repair, cell cycle arrest and apoptosis.^1–3^ Mutations of the p53 gene are observed in more than 50% of human tumor tissues, and they are considered to be the major pathogenic factor for human tumors.^4–6^ Once a p53 gene mutates it loses the regulation of various physiological activities of cells as a result of its spatial conformation, and ultimately the p53 gene is transformed from a tumor suppressor gene into an oncogene. The p53 protein-mediated cell signal transduction pathway plays an important role in regulating cellular activity and is involved in the regulation processes of genes.^1,3,7^ Therefore, it is particularly necessary to realize real-time and quantitative monitoring for p53-related diseases. The early diagnosis and prediction of a tumor can be made by monitoring the p53 gene and mutations in p53 and their products.^3,7–10^

Nucleic acid detection and quantification are playing a crucial role in molecular biology and clinical diagnostics due to their specificity and efficiency. Compared to other nucleic acid detection methods, such as DNA sequencing, DNA microarrays and electrochemistry,^11–13^ fluorescence detection has been used more widely because of its advantages of simplicity, low cost and real-time. However, traditional fluorescent probes that are used for signal transduction usually require a pre-labeled signal source, such as organic dyes or metal quantum dots (QDs), which not only suffers from a tedious operating process and high cost but also has an impact on the interaction of probes with targets.^14–16^ To make up for these deficiencies, the development of an ultra-small, simple and effective fluorescence label is in full swing.

In the presence of stabilizers, certain noble metal ions can be reduced to ultra-small nanoclusters (NCs) that are composed of several to hundreds of noble metal atoms. Since the particle size of the nanoclusters (around 2 nm or less) approaches the Fermi wavelength of electrons, they possess unique optical and electronic properties.^17–19^ When excited by light, the nanoclusters present intense light absorption and size-dependent fluorescence emission resulting from electronic transitions between discrete energy levels.^20,21^ DNA-Ag NCs (Ag NCs synthesized by using DNA as a template), an excellent fluorophore material, have great prospects in practical applications, such as biosensing and bioimaging, which will benefit from their advantages of tunable fluorescence emission (ranging from visible to near-IR), facile synthesis, high fluorescence quantum yields, low toxicity, good photostability and biocompatibility.^22–25^ Herein, enlightened by the above facts we develop a label-free fluorescent nucleic acid probe for p53 gene biosensing, where the sensing mechanism is the target-triggered fluorescence enhancement based on the generation of luminescent Ag NCs.

## 2. Experimental details

### 2.1 Materials and methods

All DNA oligonucleotides used in our work were supplied by Sangon Biotechnology Co., Ltd. (Shanghai, China) and are listed in Table S1.† Silver nitrate (99.8%, AgNO_3_), sodium borohydride (98%, NaBH_4_), and magnesium nitrate [99.0%, Mg (NO_3_)_2_·6H_2_O] were purchased from Aladdin Bio-chem technology Co., Ltd. (Shanghai, China). 20 mM phosphate buffer solution (PBS) at pH 7.0 was used throughout. All solutions were prepared with Milli-Q water (18.2 MΩ).

### 2.2 Apparatus

UV-vis absorption spectra were obtained by a Cary 50 Bio spectrophotometer (Varian Inc., CA). Fluorescence spectra were recorded on a Cary Eclipse Fluorescence Spectrophotometer (Varian Inc., CA) and a Fluoromax-4 Spectrofluorometer (HORIBA Jobin Yvon Inc., France) with 5 nm slit widths for excitation and emission at room temperature. A FL920 fluorescence lifetime spectrometer (Edinburgh Instruments, Livingston, UK) equipped with a 405 nm pulsed laser as the excitation source operating in the time-correlated single photon counting (TCSPC) mode is used to measure the luminescence decay curves, and the commercial software supplied by Edinburgh Instruments was run for data analysis. A JEOL JEM-2100 high resolution TEM instrument operating at 200 kV was employed to characterize the sizes and uniformity of the DNA-Ag NCs.

### 2.3 Synthesis of DNA-Ag NCs

In accordance with the molar ratio of Ag^+^ : NaBH_4_ : DNA = 6 : 6 : 1,^26,27^ 15 μM AgNO_3_ was added into 2.5 μM DNA solution prepared in advance in 20 mM PBS with 5.0 mM Mg^2+^ at pH 7.0. After incubating for 15 min at 4 °C in the dark, freshly prepared 15 μM NaBH_4_ was introduced into the above mixture solution and shaken vigorously for 30 s. Then the ultimate solution was further incubated at 4 °C in the dark for 2 h to synthesize Ag NCs. Unless otherwise noted, the concentration of the as-prepared DNA-Ag NCs was same as that of the above solution.

### 2.4 Agarose gel electrophoresis analysis

Three sets of sample solutions (p53 probe, p53 probe/p53, p53 probe/p53-Ag NCs) were prepared in advance. Then 8 μL of each sample was thoroughly mixed with 2 μL of loading buffer, respectively. Finally, 8 μL of the mixed sample was added into the prepared gel sample holes. A 1.5% agarose gel electrophoresis analysis of the DNA samples was operated in pH 7.0 TAE buffer at a constant voltage of 90 V for 45 minutes at room temperature. Finally, the gel was scanned by an Alpha Imager (USA).

### 2.5 Detection of target gene

In the sensing detection, different concentrations of the p53 gene were individually added into the 2.5 μM processed probe solution by heating at 85 °C for 15 min and gradually cooling to room temperature. The obtained DNA solution was incubated with gentle shaking for 2 h at room temperature to form the duplexes. Next, the synthesis of the Ag NCs is completely referenced to the steps of Section 2.3. In addition, either the single-base mismatched DNA or two-base mismatched DNA was added in place of the target DNA with the same experimental process to evaluate the selectivity of the probe. After that the fluorescence or other measurements was performed immediately.

### 2.6 Detection of target DNA in fetal bovine serum

The fetal bovine serum was diluted 100 times with PBS. The target DNA was titrated in the 1% fetal bovine serum sample following the same steps as those in PBS.

## 3. Results and discussion

**Scheme 1:**
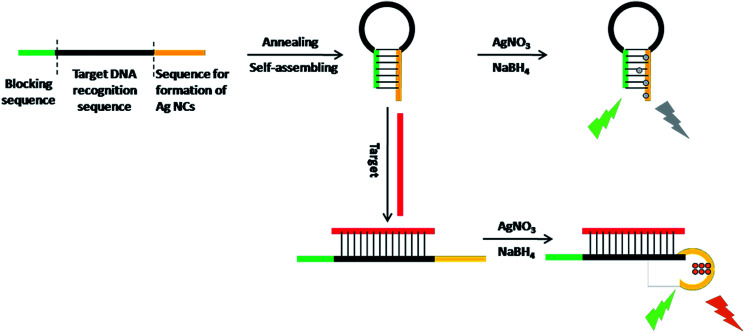
Schematic representation of the turn-on fluorescence analysis of target based on DNA-Ag NCs.

The working principle is illustrated in Scheme 1. *Via* target-responsive structural transformation of the hairpin probe, the DNA-Ag NCs are synthesized and light up the fluorescent signal. Many theoretical studies have demonstrated that the cytosine base (C) has a stronger binding affinity to Ag^+^ than the other bases, and C-rich DNA sequences can provide a favorable environment for the synthesis of Ag NCs with excellent properties.^28,29^ Thus the C-rich single-strand DNA (ssDNA) is preferentially considered as the synthetic template for Ag NCs.^30,31^ The specially designed probe contains three domains: the C-rich sequence for synthesis of Ag NCs (orange domain), the recognition sequence for the target (black domain), and the blocking sequence that is partially complementary to the C-rich sequence (green domain). When it is annealed in PBS, the random probe in the normal state will self-assemble into the locked hairpin structure as a result of the base pairs between the blocking segment and the Ag NC synthetic sequence. In the absence of a synthetic template, the Ag atoms are particularly easy to aggregate irreversibly to form larger nanoparticles without fluorescence properties.^32,33^ In contrast, if the target is introduced at this time, the C-rich DNA sequence will be freed from its locked patterns due to the complete complementary pairing between the target and the recognition sequence on the probe. Ultimately, the strong fluorescence of the as-synthesized DNA-Ag NCs provides an output signal and the detection of the target is successfully realized.

### 3.1 Characterization of DNA-Ag NCs

A great number of studies demonstrated that the synthesis of DNA-Ag NCs is strictly sequence-dependent. The fluorescence properties of DNA-Ag NCs are highly dependent on the base sequence, length and secondary structure of the DNA template.^34–37^ In our work, three common C-rich ssDNA sequences are selected for the design of the probe and they correspond to p53 probes 1, 2, and 3, respectively. Meanwhile, the other p53 probes 1–2, 2–2 and 3–2 are derived from the positional exchange of the 3′-terminus and the 5′-terminus of p53 probes 1, 2, and 3 (as marked in Table S1†), respectively. Because p53 probe 1 shows a better fluorescence effect than the other five probes in the response to the target (Fig. S1†), we eventually selected it as the detection probe for target p53.

**Fig. 1:**
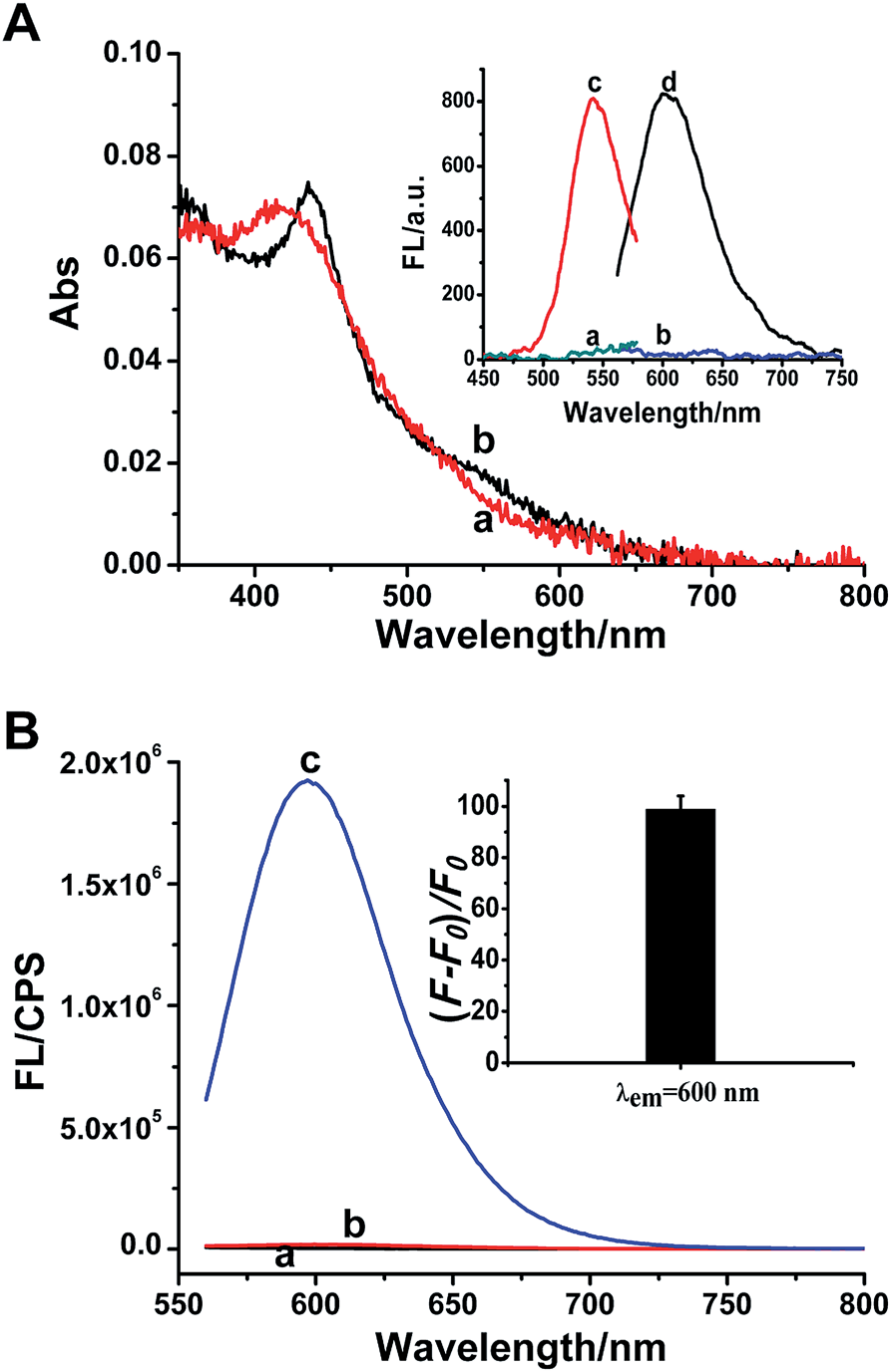
(A) UV-vis absorption spectra of the synthesized silver species based on the p53 probe 1 (curve a), or p53 probe 1/p53 (curve b). The inset shows fluorescence excitation and emission spectra of the p53 probe 1-Ag NCs (curves a and b) and the p53 probe 1/p53-Ag NCs (curves c and d). (B) Fluorescence emission spectra of DNA-Ag NCs under different conditions: (a) only p53 gene; (b) only p53 probe 1; (c) p53 probe 1/p53. The inset shows the relative fluorescence intensity (*F* − *F*_0_)/*F*_0_ recorded in the response of the p53 gene to p53 probe 1. *F* and *F*_0_ represent the emission intensity of Ag NCs in the presence and absence of p53, respectively. Error bars are calculated from three parallel experiments.

In the presence of p53 probe 1, due to the complementary pairing between its 3′-terminus and 5′-terminus, the C-rich DNA sequence that is used for synthesizing the Ag NCs is locked into a hairpin structure during the annealing process. Thus when the AgNO_3_ and NaBH_4_ are introduced, the reduced silver atoms aggregate to form larger silver nanoparticles instead of Ag NCs. As indicated in Fig. 1A, the only strong absorption peak observed at ∼420 nm (curve a) is the plasmon resonance absorption of the larger Ag nanoparticles,^38,39^ and the very weak fluorescence excitation and emission of Ag NCs recorded under this situation are shown in Fig. 1A inset (curves a and b). However, once the target p53 has been introduced into p53 probe 1, the hairpin structure of the probe is opened to release the C-rich DNA sequence, thus the fluorescent Ag NCs can be successfully synthesized. Therefore, another new absorption peak at 540 nm is observed apart from the absorption peak of silver nanoparticles at ∼420 nm (curve b, Fig. 1A), which is assigned to the characteristic peak of ultrasmall Ag NCs.^15,38,40^ Meanwhile, the as-synthesized DNA-Ag NCs also exhibit a particularly strong fluorescent emission at ∼600 nm (curve d) upon excitation at 540 nm (curve c) (Fig. 1A inset). We can even observe that the colours of the p53 probe 1-Ag NCs and the p53 probe 1/p53-Ag NCs solutions are pale yellow (left) and light red (right) (Fig. S2A†), respectively. Meanwhile, no fluorescent emission is observed for the probe 1-Ag NC solution (left) while the probe 1/p53-Ag NC solution presents a bright red fluorescence (right) when excited by a UV lamp (Fig. S2B†). According to the fluorescence emission spectra recorded in different situations, we can learn that the probe or the target itself (curves a and b, Fig. 1B) fails to play a positive role in the synthesis of Ag NCs. However, the as-synthesized Ag NCs exhibit about a 100-fold enhancement in fluorescence when the target DNA is added into p53 probe 1 (curve c and inset in Fig. 1B), which indicates that the hairpin structure of p53 probe 1 can be successfully opened by the target DNA and throw off the C-rich sequence to prepare fluorescent DNA-Ag NCs. In this way, the feasibility of our sensing strategy is verified.

A lot of research has indicated that not only the synthetic template but also the experimental conditions, such as pH and incubation time, play a vital role in the synthesis of Ag NCs. To improve the sensitivity of the biosensor, both the incubation time with NaBH_4_ and the pH of the buffer solution are optimized on the basis of the relative fluorescence intensity (*F* − *F*_0_)/*F*_0_. As shown in Fig. S3,† the probe has the best sensing performance when incubated with NaBH_4_ for 2 h at pH 7.0. Thus all the subsequent experiments are carried out under these optimum conditions.

**Fig. 2:**
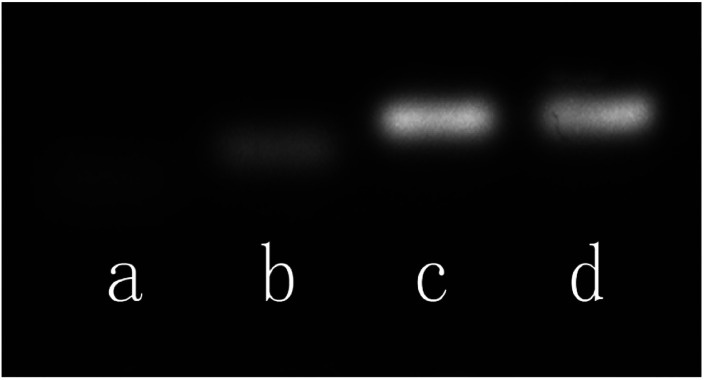
Agarose gel electrophoresis analysis of the behavior of DNA strands. Lane a: p53; lane b: p53 probe 1; lane c: p53 probe 1/p53; lane d: p53 probe 1/p53-Ag NCs.

An agarose gel electrophoresis analysis is carried out to demonstrate the target-responsive sensing mechanism. As shown in Fig. 2, the p53 probe 1/p53 (lane c) exhibits a slower migration rate than that of the target p53 (lane a) or probe 1 alone (lane b), which indicates that the target and the hairpin probe did form a stable duplex structure and then unlocked the synthetic template for Ag NCs. Meanwhile, both p53 probe 1/p53-Ag NCs (lane d) and p53 probe 1/p53 (lane c) have one and only one electrophoresis band at the same position, suggesting that the growth of Ag NCs based on a DNA scaffold will not affect the stability of the already formed duplex structure.

**Fig. 3:**
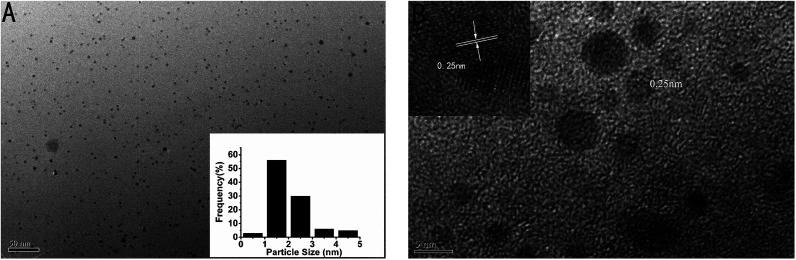
(A) TEM image of as-prepared p53 probe 1/p53-Ag NCs with an average diameter of 2 nm (inset: the size distribution histograms of the DNA-Ag NCs); (B) high-resolution TEM image of p53 probe 1/p53-Ag NCs (inset: the amplified image).

In addition, the transmission electron microscope (TEM) image of the as-prepared p53 probe 1/p53-Ag NCs was also investigated (Fig. 3A). It can clearly be observed that the as-prepared Ag NCs show good dispersity, uniformity, and a narrow size distribution with an average size of ∼1–2 nm (the inset is the size distribution histogram) when the target is added. The crystallographic structures of the as-synthesized Ag NCs are also illustrated by the HRTEM image (Fig. 3B), which not only shows that the nanoparticles in the TEM diagram are Ag NCs, but the lattice structure further illustrates the luminescence mechanism of Ag NCs. The characteristic 0.25 nm lattice fringes just correspond to the crystal lattice spacing of Ag and the continuous, single directional small domains in the Ag NCs are similar to the discrete energy levels that allow optical transitions. So the fluorescence of the Ag NCs originates from the electronic transition between these small domains whose size is comparable to the electron Fermi wavelength (approx. 0.5 nm).^17,21,41^

As demonstrated in Fig. S4,† the time-correlated single photon counting technique (TCSPC) is used to study the fluorescence lifetime of DNA-Ag NCs. The fluorescence decay is fitted with a multi-exponential (*n*) function, 
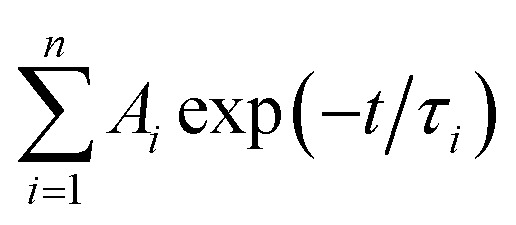
, where *A*_*i*_ are the weight percentages of the decay components with time constants *τ*_*i*_. The average excited state lifetime is expressed by the equation: 
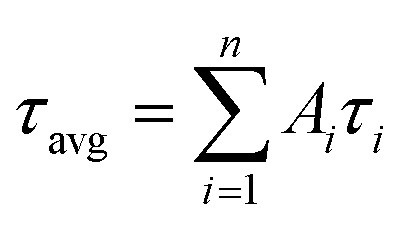
, when 
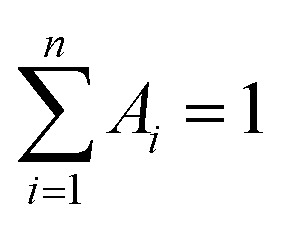
 and the results obtained for lifetime are listed in Table S2.† The bright p53 probe 1/p53-Ag NCs have a longer average lifetime (2.34 ns) than the dark p53 probe 1-Ag NCs (1.99 ns).

### 3.2 Selectivity and sensitivity

**Fig. 4:**
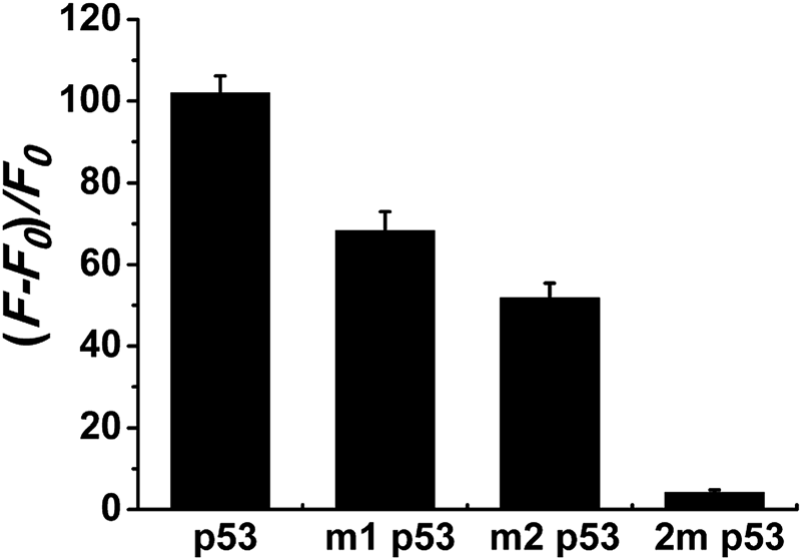
Selectivity investigation of the proposed strategy for target detection. Relative fluorescence intensity (*F* − *F*_0_)/*F*_0_ is recorded under the same experimental conditions, where *F* and *F*_0_ represent the emission intensity of Ag NCs in the presence and absence of target DNA or single-base mismatch DNAs, respectively. Error bars are calculated from three parallel experiments.

Selectivity is an important index with which to evaluate the performance of a sensor. So, we randomly selected two single-base mismatched nucleotides to investigate the sequence specificity of this probe. As shown in Fig. 4, the fluorescence responses of p53 probe 1 to two single-base mismatched sequences, m1 p53 and m2 p53, [(*F* − *F*_0_)/*F*_0_] are only 67% and 51%, respectively, which are much lower than those of the target p53 under the same experimental conditions. This result indicates that this analysis strategy has a high selectivity toward the target DNA, and even one mismatched nucleotide can be distinguished.

**Fig. 5:**
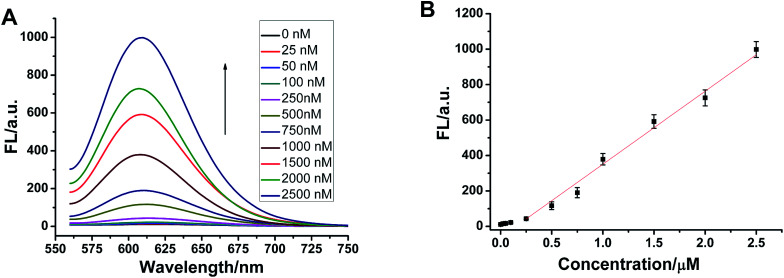
(A) The fluorescence emission spectra of DNA-Ag NCs are recorded with different concentrations of target p53. The concentration of the p53 probe DNA used is always 2500 nM. (B) The linear range is from 250 to 2500 nM between the fluorescence intensity and the concentration of target. The error bars are obtained from three independent experimental results.

To demonstrate that such a sensing strategy can be successfully applied to the quantitative analysis of the p53 gene, the fluorescent responses of the probe to different concentrations of target DNA are investigated. As expected, in Fig. 5A, the fluorescence intensity at 600 nm of Ag NCs increases gradually with an increase in concentration of target p53 from 0 to 2500 nM. Good linearity occurs in the range from 250 to 2500 nM (*R*^2^ = 0.988) (Fig. 5B). The detection limit (3*σ*/*K*, where *σ* is the standard deviation of the blank sample, *K* is the slope of the calibration curve) is estimated to be 3.57 nM. Although compared with other methods, such as electrochemistry and fluorescence amplification, the detection limit of our work is slightly high (Table S3†), the fluorescence method based on DNA-Ag NCs in our work is simple, less costly and rapid.

### 3.3 Detection of target gene in fetal bovine serum

To explore the sensing performance of the proposed probe in a complex biological medium, the recovery of the response of the target to the probe is also studied. As shown in Fig. S5B,† the fluorescence intensity of the synthesized Ag NCs in fetal bovine serum is weaker than in buffer solution, because the change in microenvironment may have an effect on the synthesis of Ag NCs or some substances in the biological medium may lead to fluorescence quenching. The fluorescence intensity at the maximum emission wavelength still increases gradually with an increase in the concentration of the target in 1% fetal bovine serum samples. The linear range was also from 250 to 2500 nM and the LOD was estimated to be 6.06 nM. These analytical results demonstrate that the designed probe can be successfully used in real biological samples.

## 4. Conclusions

In conclusion, we developed a novel hairpin DNA probe based on Ag NCs for the turn-on fluorescence sensing detection of the tumor suppressor gene p53. The probe is just a simple single-stranded DNA, but target recognition and the fluorescent signal generation are integrated into a single process because of the elegant design. The synthesized silver nanoclusters will provide a detection signal on the basis of the specific response of the probe to the target, which avoids a complex and expensive signal source labeling procedure and the possible impact of an external label on the interactions of the probes with the targets. More importantly, the novel approach can also be conveniently used for the versatile detection of diverse targets as long as the aptamer sequences of the probe are replaced accordingly. The probe may even have great potential in the simultaneous detection of multiple nucleic acids, and this probe design provides a simple, flexible and specific sensing strategy for biosensing and bioimaging.

## Conflicts of interest

There are no conflicts to declare.

## Supplementary Material

RA-008-C8RA04716B-s001
