# Supplementary material for: Nucleic acid probe based on DNA-templated silver nanoclusters for turn-on fluorescence detection of tumor suppressor gene p53
Source: RSC Adv. 2018 Jul 18;8(45):25611–6. doi: 10.1039/c8ra04716b (PMC9082775; doi:10.1039/c8ra04716b)
Supplement: RA-008-C8RA04716B-s001 [file RA-008-C8RA04716B-s001.pdf]

Electronic Supplementary Material (ESI) for RSC Advances.
This journal is © The Royal Society of Chemistry 2018

Advances

Electronic Supplementary Material (ESI) for Advances.

This journal is © The Royal Society of Chemistry 2017

Supporting Information

The nucleic acid probe based on DNA-templated silver nanoclusters for turn-on fluorescence detection of tumor suppressor gene p53

Dan Han, Chunying Wei*

Key Laboratory of Chemical Biology and Molecular Engineering of Ministry of Education, Institute of Molecular Science, Shanxi University, Taiyuan 030006, P. R. China.

*Corresponding author: Chunying Wei; E-mail address: weichuny@sxu.edu.cn.
Phone: (86) 351-7010699; Fax: (86) 351-7018429

Table S1. Names and sequences of all the DNAs used in the experiment

DNAs	Sequences (5'-3')
**p53 probe 1**	CCCTCTTAACCCGAGTCTTCCAGTGTGATGAGGGTT
**p53 probe 2**	CCCTAACTCCCCGAGTCTTCCAGTGTGATGAGGGGAG
**p53 probe 3**	CCCTTAATCCCCGAGTCTTCCAGTGTGATGAGGGGAT
**p53 probe1-2**	AAGAGGGGAGTCTTCCAGTGTGATGACCCTCTTAACCC
**p53 probe2-2**	TTAGGGGAGTCTTCCAGTGTGATGACCCTAACTCCCC
**p53 probe3-2**	TTAAGGGGAGTCTTCCAGTGTGATGACCCTTAATCCCC
**p53**	TCATCACACTGGAAGACTC
**m1 p53**	TCATCACACTGGAAAACTC
**m2 p53**	TCATCACACTGGAAGACTA
**2m p53**	TCAACACACTGGAAAACTC

Table S2. The lifetimes of DNA-Ag NCs before and after the interaction between probes and targets

P53	τ_1 (ns)	α_1 (%)	τ_2 (ns)	α_2 (%)	τ_3 (ns)	α_3 (%)	τ_{avg} (ns)	χ^2
P53 probe 1-Ag NCs	0.22	49.79	2.28	36.7	7.72	13.51	1.99	1.159
P53 probe 1-p53/ Ag NCs	1.14	20.76	2.53	77.49	8.37	1.76	2.34	1.028

This journal is © The Royal Society of Chemistry 20xx

Please do not adjust margins

Table S3. Comparison of various fluorescent methods for p53 gene detection

Methods	Linear range	Limit of detection	Reference
Colorimetric detection	—	10 nmol	[1]
Fluorescence	15 nmol – 750 nmol	4 nmol	[2]
Fluorescence	100 pM – 40 nM	1 pM	[3]
Fluorescence	—	0.07 fM	[4]
Electrochemiluminence	0.1 nmol –15 nmol	0.03 nmol	[5]
Electrochemiluminescence	0.2 pM – 200 nM	0.1 pM	[6]
Electrochemiluminence	0.001 – 0.01nM	0.68 nM	[7]
Quartz crystal microbalance	0.5 nM – 20 nM	0.3 nM	[8]

References

1 H. X. Li, L. Rothberg, P NATL ACAD SCI USA, 2004, 101, 14036.
2 R. H. Yang, J. Y. Jin, Chen Y., N. Shao, H. Z. Kang, Z. Y. Xiao, Z. W. Tang, Y. R. Wu, Z. Zhu, W. H. Tan, JACS, 2008, 130, 8351.
3 H. Xu, C. Xue, R. Zhang, Y. Chen, F. Li, Z. Shen, L. Jia, Z. S. Wu, Sens. Actuators, B, 2017, 243, 1240.
4 X. Li, J. Song, Q. W. Xue, H. Y. Zhao, M. Liu, B. L. Chen, Y. Liu, W. Jiang and C. Z. Li, Analyst, 2017, 142, 3598.
5 Y. Liu, X. Q. Chen, Q. Ma, Biosens. Bioelectron., 2018, 117, 240.
6 X. Wang, X. Zhang, P. He, Y. Fang, Biosens. Bioelectron., 2011, 26, 3608.
7 J. B. Raoof, R. Ojani, S. M. Golabi, E. Hamidi-Asl, M. S. Hejazi, Sens. Actuators B, 2011, 157, 20.
8 D. Wang, W. Tang, X. Wu, X. Wang, G. Chen, Q. Chen, N. Li, F. Liu, Anal. Chem., 2012, 84, 7008.

Please do not adjust margins

Please do not adjust margins

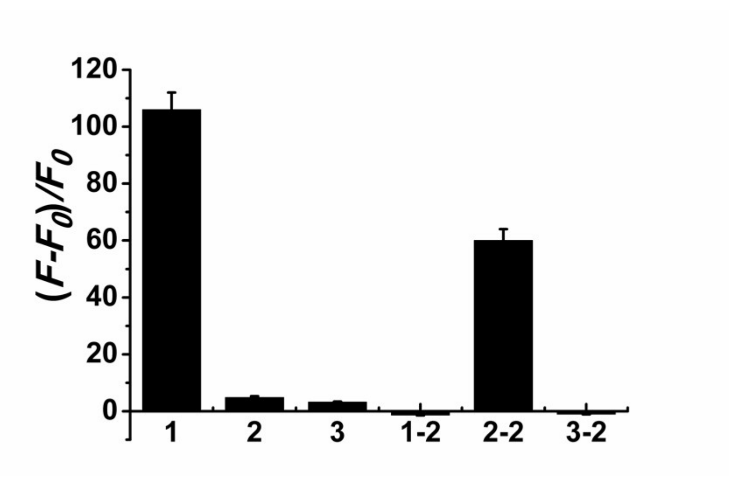

Figure S1. The relative fluorescence intensity $(F\text{-}F_0)/F_0$ is recorded by the response of p53 gene to p53 probe 1, 2, 3, 1-2, 2-2 and 3-2. *F* and F_0 represent the emission intensity of Ag NCs in the presence and in the absence of p53, respectively. Error bars are calculated from three parallel experiments.

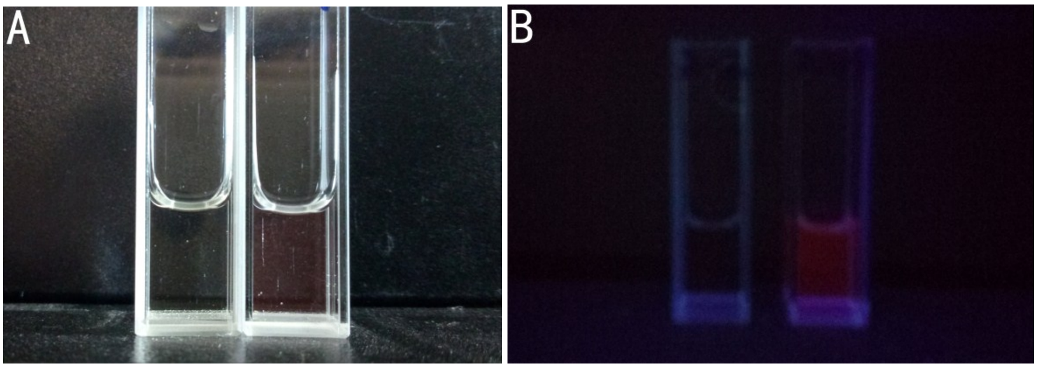

Figure S2 The corresponding photographs of p53 probe 1-Ag NCs (left) and p53 probe 1/ p53-Ag NCs (right) under the room light (A) and hand-held UV lamp (B) irradiation (365 nm), respectively.

This journal is © The Royal Society of Chemistry 20xx

Please do not adjust margins

Please do not adjust margins

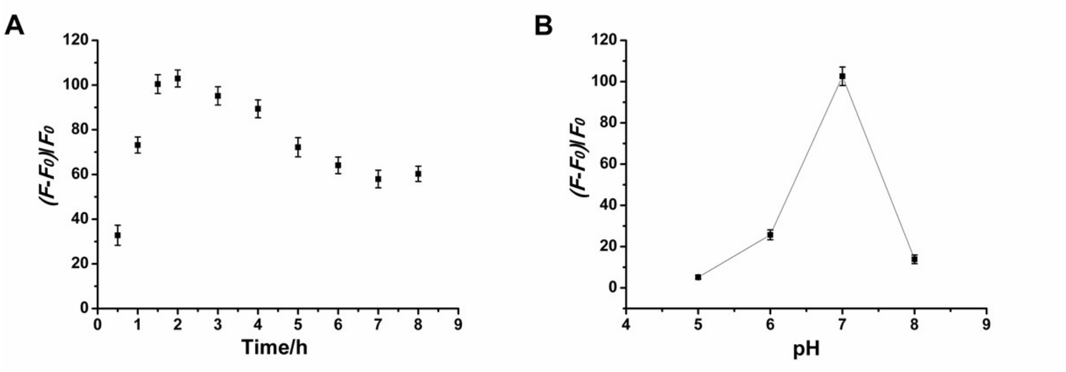

Figure S3 Effects of (A) the incubation time with $NaHB_4$ and (B) the pH value of buffer solution on the synthesis of DNA-AgNCs. The error bars are standard deviations of three repetitive measurements.

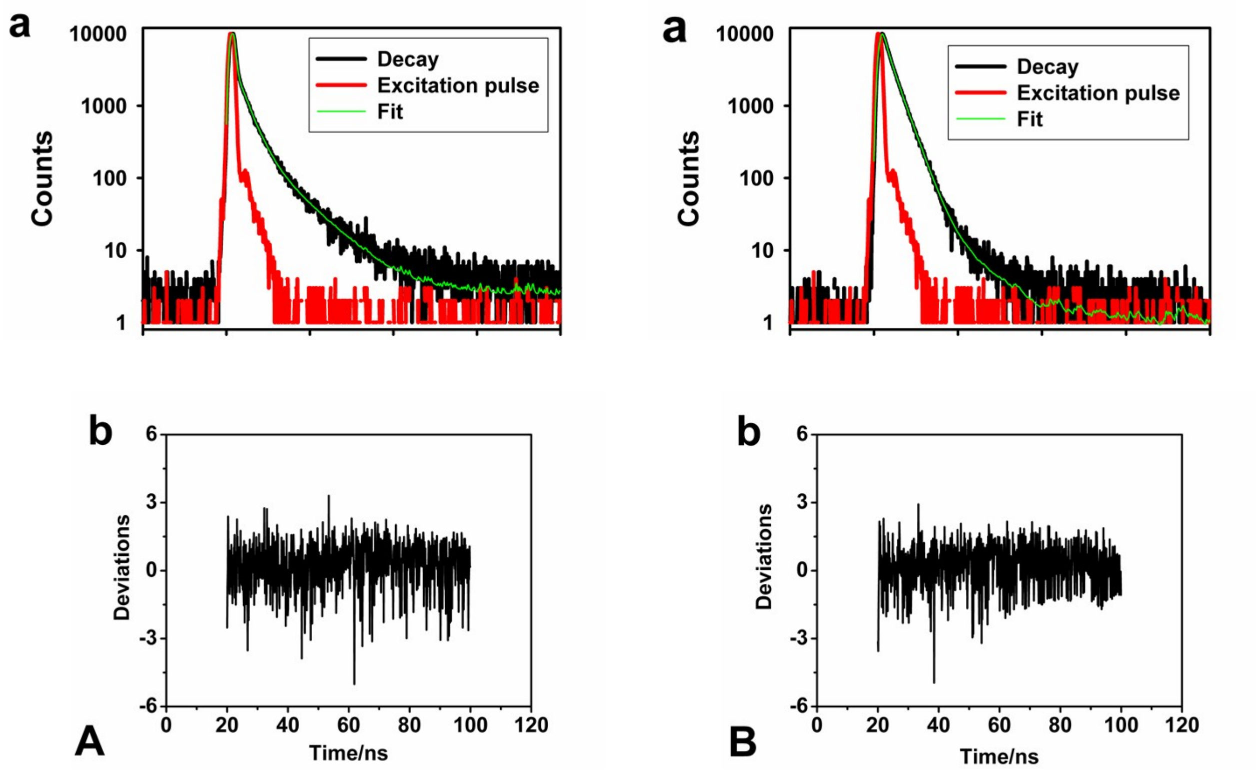

Figure S4 The TCSPC data for p53 probe 1-Ag NCs (A) and p53 probe 1/p53-Ag NCs (B), (a) the response of the instrument (red curve), DNA-Ag NCs (black curve) and the fitted curve (green curve), (b) The weighted residuals time scan of the fitted curve (excitation at 405 nm and emission at 600 nm).

Please do not adjust margins

Please do not adjust margins

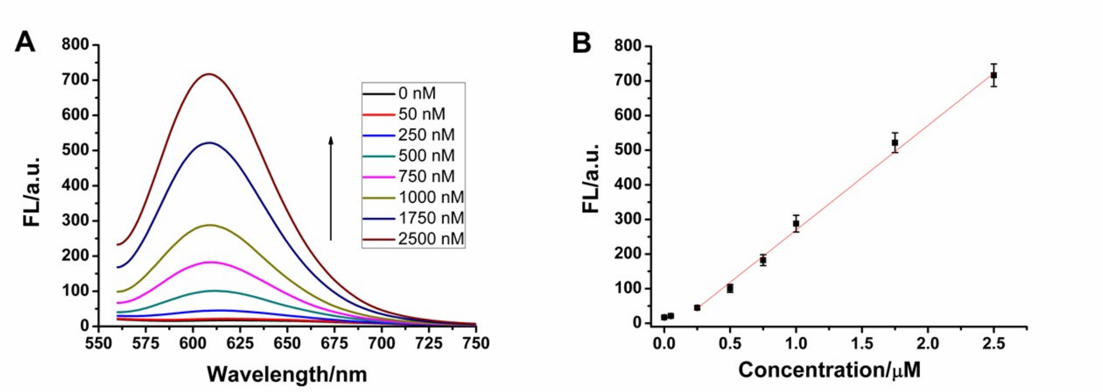

Figure S5 (A) The fluorescent emission spectra of DNA-Ag NCs synthesized in 1% fetal bovine serum samples are recorded with different concentrations of target p53. The concentration of the p53 probe 1 used is all 2.5 μM. (B) The relationship between the fluorescence intensity and the concentration of target. (C) The linear relationship between the fluorescence intensity and the concentration of p53 gene ranging from 250 to 2500 nM. The error bars are obtained according to three independent experimental results.

This journal is © The Royal Society of Chemistry 20xx

Please do not adjust margins
